# Developmental Learning Disorders: From Generic Interventions to Individualized Remediation

**DOI:** 10.3389/fpsyg.2015.02053

**Published:** 2016-01-12

**Authors:** David Moreau, Karen E. Waldie

**Affiliations:** Centre for Brain Research, School of Psychology, The University of AucklandAuckland, New Zealand

**Keywords:** developmental learning disorders, dyslexia, ADHD, cognitive remediation, neural correlates, fMRI, genetics, training interventions

## Abstract

Developmental learning disorders affect many children, impairing their experience in the classroom and hindering many aspects of their life. Once a bleak sentence associated with life-long difficulties, several learning disorders can now be successfully alleviated, directly benefiting from promising interventions. In this review, we focus on two of the most prevalent learning disorders, dyslexia and attention-deficit/hyperactivity disorder (ADHD). Recent advances have refined our understanding of the specific neural networks that are altered in these disorders, yet questions remain regarding causal links between neural changes and behavioral improvements. After briefly reviewing the theoretical foundations of dyslexia and ADHD, we explore their distinct and shared characteristics, and discuss the comorbidity of the two disorders. We then examine current interventions, and consider the benefits of approaches that integrate remediation within other activities to encourage sustained motivation and improvements. Finally, we conclude with a reflection on the potential for remediation programs to be personalized by taking into account the specificities and demands of each individual. The effective remediation of learning disorders is critical to modern societies, especially considering the far-reaching ramifications of successful early interventions.

## Introduction

Learning disorders such as dyslexia and attention-deficit/hyperactivity disorder (ADHD) represent significant challenges for children, parents, and educators. These neurodevelopmental disorders cannot be explained by intellectual ability or an inadequate learning environment, but instead appear to be due to differences in underlying brain function (Lyon et al., 2003; Nicolson and Fawcett, 2008). Combined, they impair learning in approximately one in five children, with devastating repercussions on numerous aspects of their lives. Much knowledge has been gained recently by studying the neural correlates of learning disorders, leading to the identification of specific neural networks that are typically altered in individuals with learning difficulties (Raschle et al., 2011), yet questions remain regarding causal links between changes in neural activity and behavioral improvement (Goswami, 2015). For example, recent evidence suggests that shifts toward normal brain activity do not necessarily lead to improved performance (Clark et al., 2014). If corroborated, this is an important finding, because it suggests that normalization of neural activity might not be the primary goal, and that instead remediation programs should focus on the implementation of adequate compensatory strategies (Waldie et al., submitted). Overall, important caveats remain in current behavioral interventions – remediation programs need to identify and target the specific needs of each individual to maximize improvement and to facilitate learning. Here, we provide an overview of the neural and behavioral mechanisms underlying dyslexia and ADHD, and discuss the comorbidity of the two disorders. We then explore current trends in cognitive remediation, and in particular the promise of ecological interventions. Finally, we conclude with a discussion of personalized regimens and highlight their potential in the remediation of dyslexia and ADHD.

## Distinct Characteristics of Dyslexia and Adhd

**FIGURE 1:**
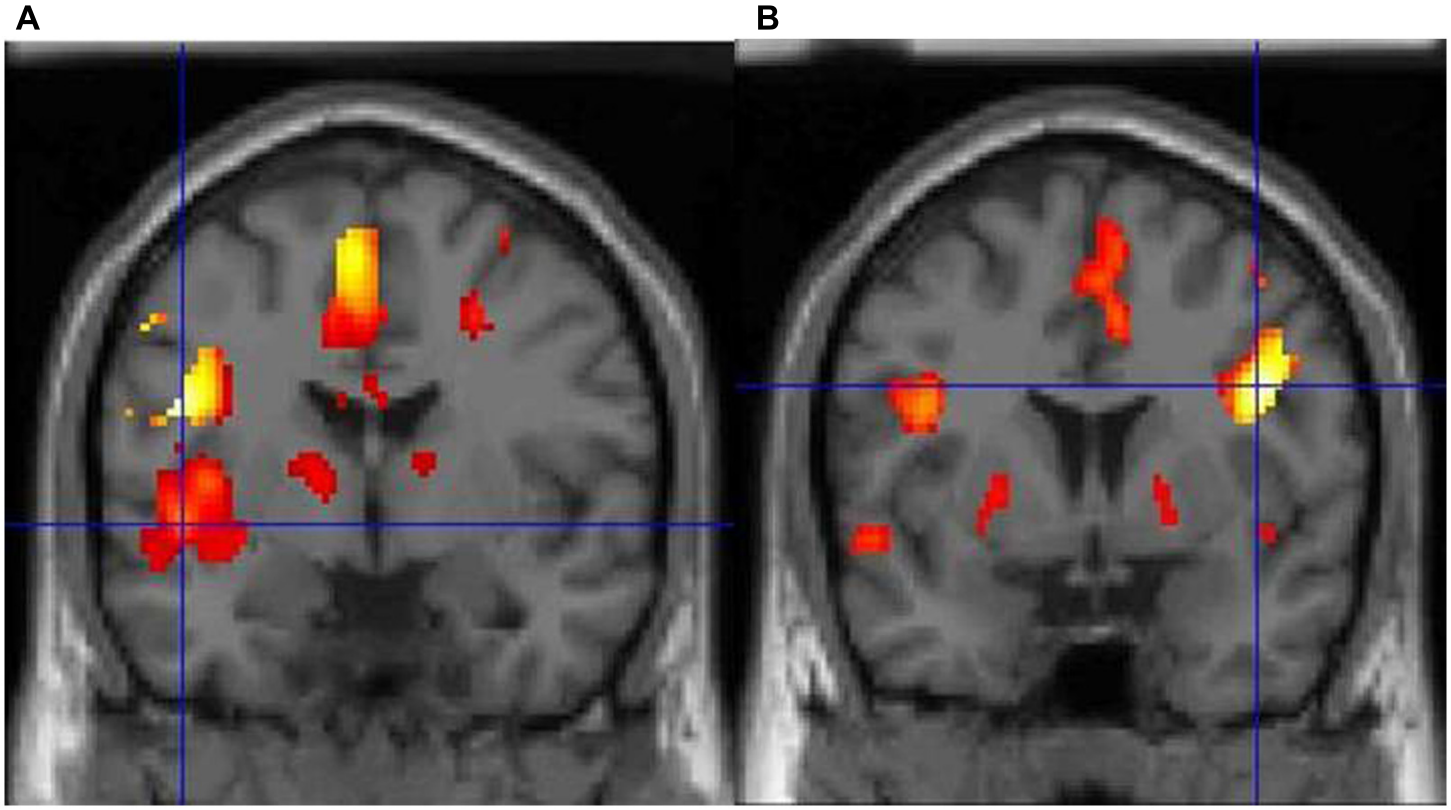
**(A)** Typical adults show a predominance of activity in the left hemisphere during lexical decision-making with concrete nouns, particularly the left posterior superior temporal cortex (marked by the crosshair). **(B)** In contrast, adults with dyslexia show very limited left-brain activity and predominant activity in the right inferior frontal cortex.

Dyslexia is primarily associated with a core speech sound (phonological) deficit (notably the inability to translate letters and letter patterns into phonological forms), with additional impairments in naming speed and working memory (Démonet et al., 2004; McCrory et al., 2005). In particular, dyslexics suffer from an inability to mentally represent words and speech sounds, or to break down complex entities into discrete sounds (Ziegler and Goswami, 2005). It should be noted that an alternative hypothesis postulates that phonological deficits emerge from visuospatial difficulties (Gabrieli, 2009; Vidyasagar and Pammer, 2010), but this is not the dominant theory currently (Skoyles and Skottun, 2009). Regardless of the cognitive bases of the disorder, dyslexia is rooted in a well-documented dysfunction of the reading network at the neural level. In particular, functional magnetic resonance imaging (fMRI) studies point toward functional and structural abnormalities in left parietal and temporal areas involved in phonological processing (Temple, 2002; Démonet et al., 2004), with compensatory engagement of anterior systems around the inferior frontal gyrus and a posterior (right occipital-temporal) system (Shaywitz et al., 2006; Waldie et al., 2013). For example, we have demonstrated that BOLD signal changes during lexical decision reveal striking differences in brain activity between typical readers and dyslexics (Waldie, 2002). The former consistently show a predominant activity in two areas of the left hemisphere, particularly in posterior (superior temporal) regions, whereas dyslexics display very limited left-brain activity, with significant activation only in the right inferior frontal cortex, probably as a compensatory system (**Figure 1**, Waldie, 2002).

Attention-deficit/hyperactivity disorder is characterized by excessive activity, short attention span and impaired inhibitory control (American Psychiatric Association [APA], 2013). Executive function deficits are common, including working memory, planning, sustained attention, inhibition, and interference control (Barkley, 1997; Overtoom et al., 2002). The main components of ADHD, attention deficit and hyperactivity, are sometimes used to differentiate between two subtypes, inattentive and hyperactive-compulsive (American Psychiatric Association [APA], 2013). Although not universal, this distinction is the most common in the scientific literature (Butterworth and Kovas, 2013). Neuroimaging studies typically show decreased functioning in the dorsolateral prefrontal cortex and dorsal anterior cingulate cortex on tasks requiring inhibitory control (Rubia et al., 2005), as well as diminished activity on inhibitory tasks in the right inferior prefrontal cortex and in the precuneus and posterior cingulate cortex (Suskauer et al., 2008). In addition, imaging studies have revealed altered patterns of activation in ventrolateral prefrontal, parietal and striatal regions (Bush et al., 2005). Despite overall consistency, there are also tremendous discrepancies in findings from this literature (Bush et al., 2005), especially magnified by methodological shortcomings (e.g., lack of control group, small sample, uncorrected multiple comparisons). Yet regardless of these limitations, studies strongly support the hypothesis that ADHD symptoms are caused by core dysfunctions in the prefrontal cortex, the basal ganglia, and by the chemical imbalance of local neurotransmitters such as dopamine, epinephrine, norepinephrine and serotonin (Aron and Poldrack, 2005).

## Comorbidity of Dyslexia and Adhd

Between 5 and 10% of the population experience severe difficulties with reading (dyslexia) or concentration/impulse control (ADHD, American Psychiatric Association [APA], 2013). While they are typically studied separately, dyslexia and ADHD occur together 30–50% of the time (Germanò et al., 2010), and only about 40% of children with dyslexia and 20% of children with ADHD present a single condition (Willcutt and Pennington, 2000a,b). Although their functional characteristics are different (Landerl et al., 2009), this line of work suggests that ADHD and dyslexia share similar underlying mechanisms (Czamara et al., 2013), and further evidence indicates that the deficits underlying both disorders are due to similar congenital neurological pathologies (Giedd et al., 1994; Waldie and Hausmann, 2010). The relation between dyslexia and ADHD might therefore be attributable to common causal influences that increase susceptibility to both disorders.

Dyslexia and ADHD share a common cognitive deficit in processing speed, and twin studies indicate that this shared weakness might arise from common genetic influences that increase susceptibility to both disorders (Willcutt et al., 2010). Shared neurological processes could also underlie reading difficulties and ADHD. In particular, a variation in cerebral lateralization related to language processing and executive functions may be a common neural mechanism. Neuroimaging studies of individuals diagnosed with dyslexia and ADHD support such a mechanism, showing reversed asymmetry of hemisphere structures among planum temporale, caudate nucleus and frontal lobes (Stefanatos and Wasserstein, 2001; Foster et al., 2002; Hoeft et al., 2007). Striatal dysfunctions have also been observed in both ADHD and dyslexia (Lou et al., 1990; Cubillo et al., 2012), and could help explain some instances of co-occurrence.

## Genetic and Environmental Risk Factors

Besides neural correlates of comorbidity, significant advances have been made in understanding the extent to which dyslexia and ADHD are attributable to genetic or environmental influences. Several family and twin studies have demonstrated that both conditions are largely heritable (Shafritz et al., 2004; Willcutt et al., 2010); however, the two conditions are polygenetic, that is, a number of genes have small additive effects that contribute specifically to each learning disorder (Plomin et al., 2008). Heritability estimates for ADHD range from 70 to 80% and from 40 to 60% for dyslexia (Shafritz et al., 2004; Del’Homme et al., 2007). Targeted linkage, association analyses and genome scans have identified potential susceptibility sites that may increase the risk of these diagnoses. In particular, genome-wide linkage analyses of dyslexia and ADHD suggest some overlap between linkage regions, which might be explained by a single gene responsible for these disorders. These regions include 1p36, 2q22-35, 3p12-q13, 4q12-13, 6p21-22, 6q12-14, 13q22-33, and 15q15-21 (Castellanos and Tannock, 2002; Gayán et al., 2005; Galaburda et al., 2006; Caspi et al., 2008; Couto et al., 2010; Faraone and Mick, 2010; Gabel et al., 2010; Scerri and Schulte-Körne, 2010).

Despite the genetic component of these learning disorders, heritable traits do not account for the full variance of their occurrence, leaving room for environmental factors and epigenetic interactions (Biederman, 2005; Ziegler et al., 2005). For example, DNA methylation and histone modification might play a critical role in these disorders, through their influence on gene regulation (Smith, 2011). Epigenetic processes guide cell differentiation and gene expression in early development, and several studies suggest that alterations can cause important cognitive deficits later in life (Gabel et al., 2010; Poelmans et al., 2011). Importantly, genetic and environmental factors may also relate to dyslexia and ADHD through vulnerability traits or unobservable characteristics of the disorders (Castellanos and Tannock, 2002), therefore obscuring observable causal mechanisms. As such, the comorbid phenotype may result from the overlap of risk factors, producing a high rate of co-occurrence. Regardless of current limitations in our understanding of these underlying mechanisms, neuroimaging and behavioral genetic studies are versatile and powerful approaches to examine the etiology and comorbidity of individual disorders – together, these studies support a partly shared genetic etiology between dyslexia and ADHD.

## Early Detection and Remediation

Advances in our understanding of both dyslexia and ADHD have allowed earlier diagnoses, and represent promising tools to inform intervention programs. Imaging findings are particularly informative in this regard, as they provide critical information about typical patterns of activity across developmental stages. For example, studies have consistently indicated that skilled reading relies primarily on a left-lateralized cortical network including frontal, temporoparietal, and occipitotemporal areas (Rumsey et al., 1997; Turkeltaub et al., 2003; Cohen and Dehaene, 2004; Pugh, 2006; Richlan, 2012), yet it is important to note that this lateralized network is preceded by more bilateral patterns of activation when learning to read (Szaflarski et al., 2006). Eventually, such bilateral activation subsides, in favor of more lateralized and efficient networks highly optimized for reading (Shaywitz et al., 2007). Similarly, executive functions are typically poor in children and adolescents (Blakemore and Choudhury, 2006), and are among the last to develop due to different rates of cortical maturation (Stiles and Jernigan, 2010). Later stages of development allow maturation of prefrontal areas (Gogtay et al., 2004), which are critical in enabling and supporting executive functions (Alvarez and Emory, 2006). Therefore, in both reading and cognitive control processes, initial neural activity can roughly be characterized as disorganized and inefficient, but eventually transitions toward better system efficiency (Laughlin and Sejnowski, 2003).

This transitional phase at the core of the reading network or the attention network is typically defective in individuals affected by dyslexia and ADHD, respectively (McCrory et al., 2005; Shaw et al., 2007). As a consequence, the use of neuroimaging techniques to identify dysfunction in these networks is a promising diagnostic tool, and such differences in neural structure and activity have been used to predict long-term outcomes in dyslexia (Hoeft et al., 2011). These neural signatures of dyslexia and ADHD also suggest that restoration of normal brain activity could alleviate or remediate these disorders. However, it is important to acknowledge that the identification of neural correlates of dyslexia and ADHD does not necessarily imply that normalizing neural activity or brain structure should be the sole focus of remediation programs, as compensatory mechanisms have also been shown to participate in behavioral improvement (e.g., Eden et al., 2004). With this limitation in mind, it remains the case that early detection has the potential to allow correcting dysfunction of these networks before they are well established and automatic. However early, accurate diagnoses do not guarantee that individuals will not experience reading or attention/hyperactivity problems if the causes are latent or immune to behavioral remediation, but this approach has the potential to be more effective than later interventions due to increased cortical plasticity in early age (Merzenich et al., 1996).

As this line or research suggests, understanding the neural bases of atypical reading and attention has important implications for remediation (Shaywitz et al., 2004). The early identification of children at risk for reading and attention deficits can help to provide them with appropriate resources and learning material. By identifying individuals whose reading or attention difficulties are the result of genetic differences in brain processing, such remediation techniques may be specifically tailored to maximize their effectiveness. Cognitive remediation programs have been shown to alleviate some of the symptoms associated with these disorders (Temple et al., 2003; Franceschini et al., 2013), but have yet to induce substantial and durable gains. Given these limitations, novel cognitive intervention paradigms combining behavioral and neurophysiological mechanisms are promising and could provide further insight into more effective remediation approaches (Diamond and Lee, 2011; Moreau and Conway, 2014).

## Ecological Approaches of Remediation

Recent trends in cognitive training have shown promises with programs that can benefit individuals in a broad manner (Diamond and Lee, 2011; Moreau and Conway, 2014). Such ecological approaches are well suited to individuals who do not present particular cognitive deficits, since they provide naturalistic environments to nurture general improvement. For example, this type of approach can allow targeting multiple components, such as cognitive gains and general health improvements in the case of regimens based on physical exercise (Tomporowski et al., 2008; Moreau, 2015). The neurobiological mechanisms underlying such improvements are well understood, and are consistent across animal and human literatures (see for a review Moreau and Conway, 2013). Likewise, seeking cognitive gains via music training has gained traction in recent years, with the added benefit of practicing an activity that is meaningful outside of the training regimen – learning to play an instrument (Kraus and Chandrasekaran, 2010; Moreno et al., 2011). Although the relevance of this approach remains to be established in the remediation of learning disorders (Bailey and Snowling, 2002), the associated changes are potentially durable, with fundamental structural changes in several cortical regions, such as the right precentral gyrus and the primary auditory region. Differences in neural activation can also be found outside auditory and motor areas, for example in bilateral frontolateral and frontomesial regions and in the left posterior pericingulate area (Hyde et al., 2009). In line with the idea of seeking cognitive enhancement through ecological means, there might even be more additional health or cognitive benefits with approaches that can be implemented outdoors (Jha et al., 2007; Berman et al., 2008; Dolgin, 2015).

Cognitive remediation represents a slightly different challenge, as it requires taking into account the specificities of the learning disorder targeted. If one’s working memory capacity is insufficient, specific training might be needed to address this limitation, and a more generic approach might lack the intensive focus required to remediate impairment. Aside from these considerations regarding content, training and remediation also have fundamental differences in terms of overarching goals – while cognitive training lacks a clear purpose due to unclear mechanisms underlying improvement (Moreau, 2014a) and potential tradeoffs in the abilities targeted (Hills and Hertwig, 2011), cognitive remediation offers an unambiguous objective: allowing the ability or set of abilities that is impaired to be improved so that it no longer impedes learning. This does not necessarily mean that the underlying mechanisms of improvement are better understood, but it allows a more direct assessment of the outcomes of an intervention. As we have mentioned previously, several studies have shown promises in the remediation of dyslexia (Temple et al., 2003) and ADHD (Klingberg et al., 2005), yet this line of work needs to be extended upon, and further replication controlling for potential confounds is required.

## Toward Individualized Regimens

What is the future of cognitive remediation programs for dyslexia and ADHD? In our view, one direction that seems inevitable is toward individualized regimens. For decades, psychologists have studied individual differences in cognition – the rationale for this entire field of research, differential psychology, is that although they share important cognitive traits, individuals differ greatly in the way their process the world around them, and that studying these differences is of importance in itself to refine theoretical models of cognition. Consistent with this idea, research on cognitive training regimens is transitioning toward an individualized approach (Moreau, 2014b; Könen and Karbach, 2015), in which individual differences are factored in to determine optimal training content. Indeed, that impairment is typically specific in dyslexia and ADHD does not mean that these specificities are necessarily consistent across all individuals diagnosed with the same disorder. For example, meta-analytic findings do not support the idea that ADHD is a disorder resulting from highly localized deficits (Dickstein et al., 2006); rather, the neural signature of ADHD is more complex, and involves individual specificities. Similar conclusions can be drawn from the study of dyslexics, who are typically identified based on behavioral manifestations, irrespective of between-individual consistency in neural substrates (Heim and Keil, 2004). As technological advances allow detecting learning impairment at an early age, remediation programs are bound to shift from a one-size-fits-all approach to individualized regimen targeting children’s specific needs and challenges.

Importantly, recognizing that individual differences matter in cognitive remediation does not legitimate the absence of empirical evidence for a particular training program – this is often the argument put forward by cognitive training corporations to justify a lack of scientific support for their claims. Rather, the rationale here is that differences across individuals need to inform training regimens to maximize outcomes (see **Table 1**). For example, software or personal tutors can use progress reports to allocate more time and resources to practice on a specific task or ability, based on clinical evidence for a particular disorder. In practice, this idea also means that a cognitive remediation program can be designed with a common structure for different learning disorders (i.e., core/general components), complemented by content tailored to the deficient or targeted abilities of each individual.

**Table 1:** Future challenges in the cognitive remediation of dyslexia and ADHD.

Goal	Means
Detection	Improve early diagnosis of dyslexia and ADHD through the combination of known risk factors and detailed mapping of neural correlates (e.g., via EEG, MEG, fMRI, DTI).
Personalization	Increase the effectiveness of remediation programs by using diagnostic data (e.g., behavioral, neural, genetic) to inform training content in a continuous manner (e.g., via Artificial Neural Networks, ANNs).
Monitoring	Assess the durability of improvements with longitudinal data collected remotely (e.g., via smartphones, tablets, wristbands, or personal computers).
Testability	Work toward building a theoretical framework of cognitive enhancement, to refine understanding of the underlying mechanisms and stability of behavioral improvement and neural changes.
Collaboration	Allow higher predictive power across individuals and research groups by sharing open-source dynamic models (e.g., online repository).

## Concluding Remarks: from Remediation to Prevention

The long-term consequences of cognitive remediation are presently unclear – in the broader field of cognitive enhancement, some have pointed out the limits of our current understanding regarding the underlying mechanisms and potential tradeoffs involved in better cognitive performance (Hills and Hertwig, 2011; Moreau, 2014a). Until we can successfully integrate findings within a theoretical framework of cognitive enhancement, cognitive remediation studies will remain a heterogeneous collection of work potentially tapping into different mechanisms. With this limitation in mind, advances in neuroscience allow detecting potential learning disorders earlier – ultimately, specific remediation programs will strive to prevent difficulties rather than remediate existing disorders. The impact of such interventions is difficult to foresee, as this approach is novel in the remediation of dyslexia and ADHD, but it has the potential to be particularly influential. In addition to clinical benefits, preventing disorders is also a more rational and efficient approach than post-diagnoses remediation, thus offering brighter outlooks to many children, as well as numerous advantages to the community.

## Conflict of Interest Statement

The authors declare that the research was conducted in the absence of any commercial or financial relationships that could be construed as a potential conflict of interest. The reviewer, Adriana Marques de Oliveira, and handling editor declared their shared affiliation, and the handling editor states that the process nevertheless met the standards of a fair and objective review. The reviewer, Vera Lúcia Orlandi Cunha, and handling editor declared their shared affiliation, and the handling editor states that the process nevertheless met the standards of a fair and objective review.
